# Contemporary incidence and risk factors for carotid artery disease in patients referred for coronary artery bypass surgery

**DOI:** 10.1186/1749-8090-7-78

**Published:** 2012-08-28

**Authors:** Kelly M Wanamaker, Robert J Moraca, Diane Nitzberg, George J Magovern

**Affiliations:** 1 Department of Cardiovascular and Thoracic Surgery, Allegheny General Hospital, Pittsburgh, PA; 2 Department of Cardiovascular and Thoracic Surgery, Allegheny General Hospital, 320 East. North Avenue, Pittsburgh, PA

**Keywords:** Coronary artery bypass surgery, Carotid artery stenosis

## Abstract

**Background:**

In the past decade, there has been an increase in the amount of patients with medical co-morbidities referred for coronary artery bypass surgery (CABG). Significant carotid artery disease in patients undergoing CABG procedures increases the risk of neurological complications. We review the results of routine carotid screening in patients undergoing CABG to determine the contemporary incidence and risk factors for carotid artery disease.

**Methods:**

Between 2008 through 2010, 673 patients were referred for isolated coronary artery bypass surgery at a single institution. Patients were identified through a systematic review of The Department of Cardiothoracic Surgery Society of Thoracic Surgery Outcomes Database. A retrospective analysis of prospectively collected demographic, clinical data and outcomes were performed. All patients with screening preoperative carotid duplex were reviewed. We defined the degree of carotid disease as: none to mild stenosis (<50%), moderate stenosis (50-69%), severe stenosis (70-99%). Multivariate analysis was performed to identify risk factors.

**Results:**

559 (83%) patients underwent screening preoperative carotid ultrasonography prior to CABG. The incidence of carotid artery disease (>50% stenosis) was 36% with 18% unilateral moderate disease, 10% bilateral moderate and 8% severe disease. Risk factors associated with carotid artery disease included: advanced age, renal failure, previous stroke, peripheral vascular disease, left main coronary artery disease, and previous myocardial infarction.

**Conclusions:**

There is a significant incidence of carotid artery stenosis in patients referred for CABG. Routine screening will identify patients with carotid artery disease and may reduce the risk of postoperative stroke.

## Background

Stroke is a deleterious complication of coronary artery bypass grafting (CABG) with an incidence of 1.2% [1]. Despite improvements in anesthesia and surgical techniques, stroke remains a devastating neurologic complication of myocardial revascularization and is one of the primary concerns when assessing a patient’s cardiopulmonary bypass candidacy.

The etiology of a cerebrovascular accident after CABG is multifactorial making it difficult to determine which mechanism is implicated in a particular event. Calcific debris from a diseased valve, macroemboli of cardiac origin, introduction of air during the procedure, hypoperfusion arising from a severely stenotic carotid artery or embolization from an ulcerated plaque have all been described [2].

Several studies have shown that the existence of carotid artery stenosis (CAS) in subjects undergoing cardiopulmonary bypass procedures increases the risk of significant neurological deficits [3-9]. Intraoperative hemodynamic instability and anemia with resultant cerebral hypoperfusion particularly in patients with extracranial carotid disease may account for a significant number of intraoperative events. Furthermore, studies have shown a direct link between degree of carotid artery stenosis and risk of ipsilateral stroke [10]. The inherently poor postoperative course of patients who develop stroke after CABG underlines the need for timely recognition and modification of factors that predispose to stroke.

The objective of our study was to investigate the contemporary incidence of CAS in patients undergoing isolated CABG and to determine risk factors related to carotid stenosis.

## Methods

### Study population

Between January 2008 and January 2010, 673 consecutive patients underwent isolated CABG at Allegheny General Hospital. Patients were identified through a systematic review of The Department of Cardiothoracic Surgery Society of Thoracic Surgery Outcomes Database. The Institutional Review Board (IRB) approved the study. A retrospective analysis of prospectively collected demographic and clinical data and clinical outcomes was performed.

### Carotid artery evaluation

559 (83.1%) patients underwent bilateral carotid duplex ultrasonography to analyze for the presence and extent of stenosis in their cerebrovasculature. Patients who did not receive preoperative carotid screening were those with either ongoing angina, hemodynamic instability in need of urgent coronary revascularization, or physician preference. Duplex measurements of peak systolic velocity (PSV) of the internal carotid artery (ICA) were recorded, and the ratio of these velocities in the internal and common carotid arteries (ICA: CCA) was calculated (Table 1). The criteria determined for detection of 50% or greater stenosis were as follows: peak systolic velocity of the internal carotid artery greater than 125 cm/s and ratio of peak systolic velocity of the internal carotid artery to peak systolic velocity of the common carotid artery greater than 2. Our definition of degree of stenosis paralleled that of radiologic guidelines [11], none to mild stenosis (<50%) is defined as PSV <125cm/sec and an ICA:CCA ratio of <2.0, moderate stenosis (50-69%) as a PSV of 125 to 229 cm/sec or an ICA:CCA ratio between 2.0 and 3.9, and severe stenosis (70-99%) as a PSV of ≥230 cm/sec or an ICA:CCA ratio ≥4.0 and a occluded carotid artery is defined as having a PSV and an ICA:CCA ratio of 0. We defined significant and severe carotid artery stenosis as >50% and >70% respectively.

**Table 1:** Carotid artery stenosis definition and degree of severity

***Duplex US Measurement***	***Mild <50%***	***Moderate 50-69%***	***Severe 70-99%***
PSV_ICA_ (mm/sec)	<125	125-229	>230
PSV_ICA_/PSV_CCA_	<2	2.0-3.9	>4.0

### Measured risk factors

The following clinical and demographic risk factors were analyzed for incidence and correlation with carotid artery disease: age, sex, congestive heart failure, hypertension, chronic obstructive pulmonary disease (COPD), history of stroke, peripheral vascular disease (PVD), hemodialysis dependent chronic kidney disease (CKD), previous myocardial infarction, body mass index, history of smoking, familial history of CAD, hyperlipidemia, diabetes mellitus, previous percutaneous coronary intervention, hyperlipidemia, left main trunk disease (LMT) and the number of coronary bypasses were examined [8,12-15].

### Statistical analysis

Our prospectively collected database was interrogated to analyze preoperative patient characteristics, risk factors and degree of carotid stenosis. They were compared by **univariate analysis** using Statistical Analysis Systems (SAS Institute, Cary, North Carolina). A p value < 0.05 was considered significant.

## Results

Significant carotid artery stenosis (>50%) was found in 200 of 559 (35.7%) of our patient population (24.3% women, mean age 67). In the 559 patients who had preoperative carotid duplex ultrasound, as listed in Table 2, 64% had mild to no stenosis and **36%** had significant carotid artery stenosis (>50%).

**Table 2:** Incidence of carotid artery disease

***Degree of Stenosis***	***CABG (n=559)***
Mild/None	359 (64%)
Stenosis >50%	200 (36%)
Unilateral Moderate	98 (18%)
Bilateral Moderate	53 (9.4%)
Unilateral Severe/Occluded	20 (3.5%)
Bilateral Moderate & Severe	15 (2.6%)
Bilateral Severe	14 (2.5%)

The clinical and demographic characteristics of patients with and without significant CAS are compared in Table 3. Logistic multiple regression analysis revealed the following independent risk factors for significant CAS: advanced age, female gender, hypertension, history of cerebrovascular accidents (CVA), dialysis, and peripheral vascular disease. Additionally, left main disease may offer some insight to the severity of atherosclerotic disease. Variables such as smoking, hyperlipidemia, and diabetes were not statistically significant.

**Table 3:** Demographic and clinical characteristics of Patients with Significant Carotid Stenosis (>50%)

	***All (n=559)***	***No CAS (n=359)***	***CAS (n=200)***	***p value***
Mean Age (years)	67	65.5	68.6	**0.003**
Women	24%	24%	32%	**0.0046**
CHF	13%	12%	17%	0.12
Hypertension	94%	94%	92%	**0.04**
COPD	23%	21%	27%	0.1
CVA	5.4%	3.8%	8%	**0.02**
PVD	15%	10%	23%	**0.0001**
CKD (Dialysis)	13%	9%	21%	**0.0001**
History of MI	45%	44%	47%	0.47
BMI (Mean)	30	30.5	30.1	0.4
History of Smoking	59%	58%	62%	0.9
Family History of Coronary Disease	33%	35%	28%	0.09
Hyperlipidemia	85%	84%	86%	0.6
Diabetes	36%	35%	40%	0.7
Previous PTCA	27%	27%	26%	1.0
Antilipid Use	71%	69%	70%	0.9
Left main Trunk	31%	27%	37%	**0.016**
Number of Bypass	2.52	2.53	2.52	0.8

CAS=Carotid artery stenosis > 50%.

Severe CAS= Carotid artery stenosis > 70% unilateral or bilateral.

Patients with severe carotid artery stenosis > 70% were compared to those without significant disease in Table 4. Congestive heart failure (CHF), history of myocardial infarction, and recent or remote smoking history were also found to be significant variables. Those factors associated with significant stenosis but not severe stenosis were female gender and hypertension.

**Table 4:** Demographic and clinical characteristics of Patients with Severe Carotid Stenosis (>70%)

***No CAS***	***Severe CAS only***	***p value***	
	**(n=359)**	**(n=49)**	
Age	65.5	69.2	**0.01**
Women	24%	24%	1.0
CHF	12%	29%	**0.003**
Hypertension	94%	92%	0.5
COPD	21%	30%	0.14
CVA	3.8%	10%	**0.05**
PVD	10%	33%	**0.0001**
CKD (Dialysis)	9%	22%	**0.01**
History of MI	44%	61%	**0.02**
BMI (Mean)	30.5	31.1	0.5
History of Smoking	58%	75%	**0.019**
Family History of Coronary Disease	35%	25%	0.15
Hyperlipidemia	84%	90%	0.39
Diabetes	35%	42%	0.34
Previous PTCA	27%	22%	0.6
Antilipid Use	69%	82%	0.09
Left main Trunk	27%	53%	**0.0004**
Number of Bypass	2.53	2.65	0.3

CAS=Carotid artery stenosis > 50%.

Severe CAS= Carotid artery stenosis > 70% unilateral or bilateral.

## Discussion

### Incidence of carotid artery stenosis in CABG patients

It has been shown that significant CAS is common in patients undergoing CABG [13]. In studies which examined all patients undergoing CABG, CAS ≥ 50% has been detected in nearly one in three patients [16]. In our study the incidence of significant carotid artery stenosis in patients referred for isolated CABG is 36%. This is consistent with previous studies which have reported a prevalence of 6.1%–31.7% in CABG patients depending upon definitions of the degree of stenosis and methods of screening [4,13,16-19]. For severe carotid stenosis (>75%), the prevalence has been reported to be 4.1%-13.3% [8,9,12,16] which also remains consistent with our findings of >70% stenosis in 8.8% of patients.

### Risk factors for carotid stenosis

With the data provided by these studies, univariate analysis identified advanced age, female gender, hypertension, prior stroke, dialysis, peripheral vascular disease (PVD), and left main disease as independent risk factors for significant carotid stenosis. Except for congestive heart failure (CHF), prior myocardial infarction (MI), and smoking predictors for severe CAS (>70%) were similar. These variables have been considered positive predictors in the genesis of atherosclerotic disease [20].

In a study by Mahmoudi and colleagues in 2011, carotid duplex ultrasound was performed on 878 patients prior to isolated CABG and they found that 13% had a carotid stenosis greater than 75% [16]. Significant predictors for CAS were age >69 and PVD. Unlike our study, CHF, prior MI and tobacco abuse were not statistically significant.

Peripheral vascular disease, end stage kidney disease, advanced age, and prior stroke have been reported by other investigators as co-morbidities associated with CAS [9,12-14]. Our study similarly showed that they are the strongest predictors of CAS.

In 2010, Drohomirecka, et al. evaluated 682 patients using duplex ultrasound and found that 123 (18%) had carotid stenosis greater than 50% and 29 (4.5%) with severe CAS [13]. Predictors of significant carotid artery disease were history of cerebrovascular accidents, PVD, unstable angina, and older age. The predictors of severe stenosis (at least one carotid artery ≥ 70%) were a history of stroke, PVD, and presence of left main disease.

Female gender has not been found to be a predictive factor for carotid stenosis in CABG patients; however, women undergoing CABG are at greater risk for major complications than men because of the comorbid conditions that are associated with the later age at which women present for coronary surgery and not because of gender [14]. In our study, the incidence of carotid stenosis >50% in female patients was statistically different (p= 0.0046) when compared to no CAS. Similarly, Durand et al. [9] and D’Agostino et al. [17] identified female gender, age >65 years, PVD, prior CVA, left main coronary disease, and hypertension as risk factors for significant carotid stenosis.

Advanced age is shown to be an independent risk factor for stroke in CABG, mainly among octogenarians [3]. Age is also an important risk factor for carotid artery disease [8,9,13,14,16]. One analysis of 1,068 patients showed 167 (15.6%) with carotid artery stenosis >50%. The prevalence of stenosis in that group increased with age. CABG patients aged <60 years had a 4% prevalence, rising to 11% in patients >60 and 15% in those aged >70 [21].

Peripheral vascular disease has been reported by other investigators as a comorbidity associated with CAS [9,13,14,19,21]. Salasidis et al. [8] found a history of peripheral vascular disease in 81 (20.9%) of 387 patients scheduled for nonemergent CABG; and among these 81 subjects, 26% (n=21) had significant carotid stenosis.

The aforementioned clinical parameters may indicate a need for preoperative carotid ultrasound examination in patients undergoing isolated coronary revascularization. Patients with concomitant cerebrovascular and coronary artery disease represent a subset with advanced atherosclerosis in which other areas of the vascular system are involved. The incidence of carotid stenosis in patients with coronary artery disease is high which can translate to an increased risk of perioperative stroke. We found several demographic and clinical risk factors that are markers for atherosclerosis which may indicate the need for preoperative screening for CAS in patients undergoing isolated CABG.

### Limitations of the study

The study was a retrospective review of medical records. Some patients undergoing urgent surgery were not included in the study due to lack of ultrasonographic examination.

## Conclusions

There is a significant incidence of carotid artery stenosis in patients referred for CABG. Routine screening will identify patients with carotid artery disease and may reduce the risk of postoperative stroke.

## Competing interests

The authors declare that they have no competing interests.

## Authors’ contributions

KMW and RJM wrote the manuscript, statistical analysis DN performed the data collection, GJM performed editorial oversight and assisted with statistical analysis. All authors read and approved the final manuscript.
